# Mathematical modeling of temperature-induced circadian rhythms

**DOI:** 10.3389/fsysb.2024.1256398

**Published:** 2024-03-25

**Authors:** Lingjun Lu, Yannuo Li, Rene Schloss, Ioannis P. Androulakis

**Affiliations:** ^1^ Chemical and Biochemical Engineering Department, Rutgers University, Piscataway, NJ, United States; ^2^ Biomedical Engineering Department, Rutgers University, Piscataway, NJ, United States

**Keywords:** circadian, systems biology, temperature, shiftwork, circadian disruption (CD)

## Abstract

The central circadian pacemaker in the suprachiasmatic nuclei (SCN) aligns the phase and period of autonomous molecular oscillators in peripheral cells to daily light/dark cycles via physiological, neuronal, hormonal, and metabolic signals. Among different entrainment factors, temperature entrainment has been proposed as an essential alternative for inducing and sustaining circadian rhythms *in vitro*. While the synchronization mechanisms for hormones such as glucocorticoids have been widely studied, little is known about the crucial role of body temperature as a systemic cue. In this work, we develop a semi-mechanistic mathematical model describing the entrainment of peripheral clocks to temperature rhythms. The model incorporates a temperature sensing-transduction cascade involving a heat shock transcription factor-1 (HSF1) and heat shock response (HSR) pathway to simulate the entrainment of clock genes. The model is used to investigate the mammalian temperature entrainment and synchronization of cells subject to temperature oscillations of different amplitudes and magnitudes and examine the effects of transitioning between temperature schedules. Our computational analyses of the system’s dynamic responses reveal that 1) individual cells gradually synchronize to the rhythmic temperature signal by resetting their intrinsic phases to achieve coherent dynamics while oscillations are abolished in the absence of temperature rhythmicity; 2) alterations in the amplitude and period of temperature rhythms impact the peripheral synchronization behavior; 3) personalized synchronization strategies allow for differential, adaptive responses to temperature rhythms. Our results demonstrate that temperature can be a potent entrainer of circadian rhythms. Therefore, *in vitro* systems subjected to temperature modulation can serve as a potential tool for studying the adjustment or disruption of circadian rhythms.

## Introduction

The circadian timing system enables organisms to anticipate daily environmental variations and adapt their physiological activities and behaviors correspondingly. In mammals, virtually every cell contains a clock with a period of ∼ 24 h , driven by a cell-autonomous molecular machinery comprised of interdependently regulated genes and proteins. This autoregulatory network arises from interlocked positive and negative transcriptional-translational feedback loops. The core negative feedback loop consists of the heterodimer CLOCK/BMAL1 binding to the E-box element of target genes *period* (*Per1*, *Per2*) and *cryptochrome* (*Cry1*, *Cry2*), activating their transcription and producing the cytoplasmic proteins PER and CRY. These gene products dimerize to form the PER/CRY complex, which in turn translocates to the nucleus and functions as an indirect repressor of their transcription by inhibiting the transcriptional activity of CLOCK/BMAL1 (Mohawk et al., 2012). In addition, the transcription of *Bmal1* is positively regulated by PERs and CRYs through downregulation of the expression of *Rev-erbα* (Reppert and Weaver, 2001; Becker-Weimann et al., 2004), which causes an antiphase oscillation of cytoplasmic BMAL1 (Mohawk et al., 2012).

These individual cells are driven by cues that synchronize their oscillations (Balsalobre et al., 2000; Damiola et al., 2000; Yamazaki et al., 2000; Stokkan et al., 2001). The cues are centrally coordinated by the master circadian clock located in the hypothalamus’s suprachiasmatic nucleus (SCN), which is primarily entrained to external light/dark cycles (Li and Androulakis, 2022). While the master clock is fairly robust, peripheral clocks are more prone to desynchronization, leading to diverse pathological conditions (Nudell et al., 2019). Several factors, including steroid hormones and (core body) temperature, have been considered potent internal entrainers in resetting peripheral clocks (Balsalobre et al., 2000; Kornmann et al., 2007; Buhr et al., 2010).

Experimental systems that can maintain or disrupt circadian rhythms are essential for studies aiming at understanding the pathophysiology of circadian disruption (Ndikung et al., 2020). While *in vivo* systems primarily use light and feeding as the key entrainers of circadian rhythms, few *in vitro* systems demonstrate the ability to sustain circadian oscillations. Circadian clocks in cultured cells can be synchronized, albeit transiently (Yamazaki et al., 2000), with a variety of signals, including serum, glucocorticoid hormones, synthetic glucocorticoids (e.g., dexamethasone), retinoic acid, Ca^2+^ ionophores, tumor promoters, and growth factors (Stratmann and Schibler, 2006) However, experimental studies have demonstrated that peripheral clocks in astrocytes, fibroblasts, liver, kidney, and lung, can be stably entrained to temperature pulses that mimic body temperature rhythms (Brown et al., 2002; Prolo et al., 2005; Abraham et al., 2010; Buhr et al., 2010; Saini et al., 2012). This offers the possibility of using temperature modulation as an *in vitro* stimulus to facilitate studies of circadian rhythms (Nudell et al., 2019). Unlike biochemical signals, external temperature rhythms can be modulcated at will and provide a more consistent way of generating circadian rhythms. In particular, this motivates the development of both experimental and theoretical systems that use temperature as a global entrainer to induce and sustain circadian expression patterns in cells.

The daily fluctuation of mammalian core body temperature lies within a narrow physiological range. It arises from a continuous interplay between the central circadian clock, the thermoregulatory center located in the preoptic hypothalamus, and heat-modulating tissues/organs (Morf and Schibler, 2013). Temperature variations are sensed by temperature-sensitive sensors throughout the body and brain (Dhaka et al., 2006; Benarroch, 2007; Romanovsky, 2007). Studies have also demonstrated the rhythmic accumulation of heat shock protein (*Hsp*) mRNA (Kornmann et al., 2007) resulting from a rhythmic activation of the transcription activator heat shock transcription factor-1 (HSF1) (Reinke et al., 2008) in response to temperature cycles. While total *Hsf1* mRNA and HSF1 protein levels do not vary diurnally, the fraction of activated nuclear HSF1 during the active phase, when body temperature is elevated, is increased, pointing to a critical regulatory role (Saini et al., 2012).

Given that HSF1 acts as the regulatory output of a wide range of cellular signaling pathways (Akerfelt et al., 2010), it likely represents a point of convergence of several regulatory pathways, including the temperature sensing-transduction (Mohawk et al., 2012). Furthermore, the resetting of *Per2* expression by circadian temperature pulses in tissue explants is blunted by the blockage of an HSF1 inhibitor (Buhr et al., 2010) and in HSF1-deficient fibroblasts (Tamaru et al., 2011). These observations suggest a signaling cascade involved in the entrainment of peripheral circadian clocks by (rhythmic) temperature signals.

In this work, we propose a mathematical model accounting for temperature as a *zeitgeber*, acting on a temperature signaling cascade entraining a population of cells. By conducting *in silico* experiments with the model, we reproduce several key experimental observations indicating that 1) the temperature rhythmicity induces the synchronization of peripheral cell clocks in an amplitude- and period-dependent manner, 2) desynchronization and synchronization of peripheral cells can be modulated by implementing appropriate temperature schedules, and 3) the ability of the system to adapt to temperature schedules depends on individual properties. Our model predicts a possible connection between a system’s abilities to maintain inherent rhythmicity and to attune to new circadian schedules. Taken together, these results demonstrate that temperature-entrained *in vitro* systems can replicate circadian dynamics observed *in vivo,* thus advancing the idea of developing more physiologically realistic *in vitro* systems for studying circadian rhythms.

## Materials and methods

**FIGURE 1:**
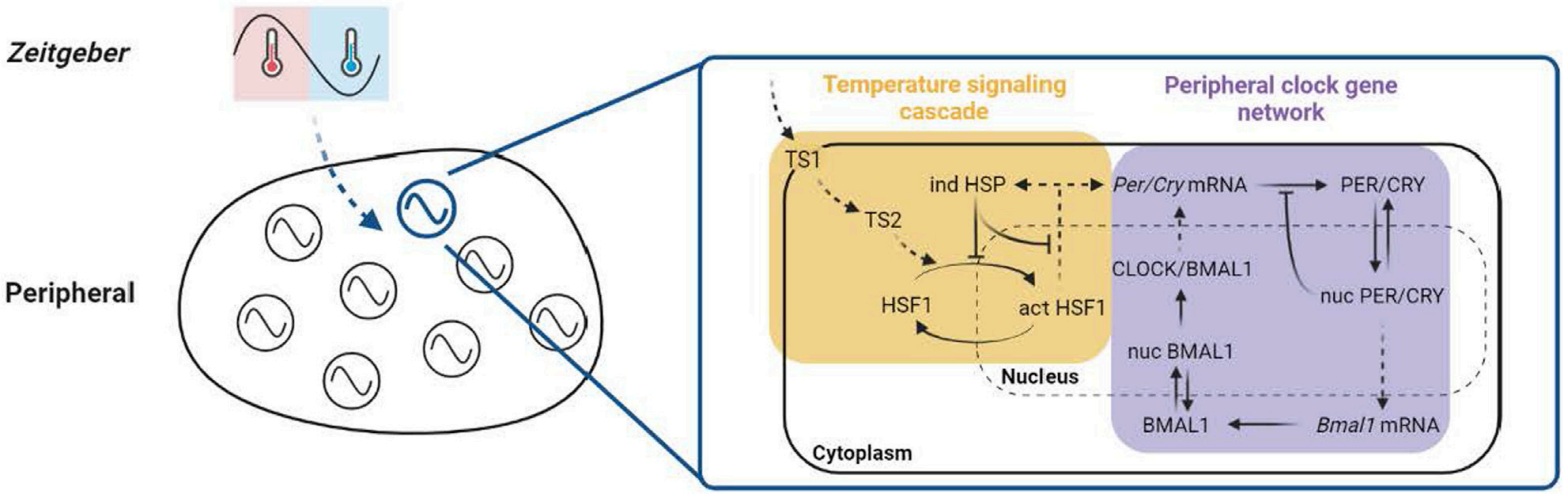
Schematic of the model. The ODE-based model is built on two sections, the temperature signaling cascade and the peripheral clock gene network using the indirect response model (IRM). In the temperature signaling cascade (yellow panel), a generalized thermosensor-transductor dynamics (*TS_1_, TS_2_*) is assumed to amplify the relative intensity of the temperature rhythm, as denoted by the ratio of the amplitude to the average of the component oscillation. This ampled temperature signal is then propagated to a downstream effector, the HSF1 -mediated HSR pathway. The temperature sensing process is hypothesized to be activated by integrating the differential sensing of temperature magnitude (represented by the average) and variation (representedby the amplitude). In addition, a classical HSR is assumed to be partly involved in the temperature entrainment by considering only the stimulatory effect of active HSF1 on the HSP production and inhibitory feedbacks of the HSF1 -induced HSP. In the peripheral clock gene network (purple panel), a gene regulatory network model consisting of positive and negative limbs is used to describe the intracellular circadian dynamics of clock genes and proteins. The figure is created with BioRender.com.

To capture the mammalian peripheral entrainment of the temperature rhythm, we propose a semi-mechanistic, ordinary differential equation (ODE)-based model consisting of two major components: a temperature signaling cascade and a prototypical peripheral clock gene network (Figure 1).

### Temperature sensing and transduction

Temperature rhythms are systemic cues that efficiently entrain individual clocks in cultured cells and tissue explants (Morf and Schibler, 2013). A possible mechanism involves the post-transcriptional regulation of the cytoplasmic abundance of *Clock* mRNA by cold-inducible RNA-binding protein (CIRBP) (Morf et al., 2012) and the transcriptional induction of the nuclear *Per2* mRNA by heat shock factor 1 (HSF1) (Saini et al., 2012). Experimental evidence shows that cold and heat exposure can activate HSF1 (Cullen and Sarge, 1997) and upregulate the nuclear Per2 transcript (Chappuis et al., 2013; Fischl et al., 2020), so we focus on the HSF1-associated mechanism in our model.

Without loss of generality, temperature rhythms are modeled as square wave functions. Such an approximation enables us to capture the essential oscillation characteristics such as the phase, period, and peak-to-trough amplitude (Eq. 1). Thermosensing and thermoregulation are closely interconnected (Xiao and Xu, 2021). Since temperature regulates nearly all the physiological processes in living organisms, thermosensors are ubiquitous and indispensable in sensing, transducing, and processing external and internal temperature fluctuations (Xiao and Xu, 2021). Thermosensitive transient receptor potential channels (thermo-TRPs) are molecular thermosensors shown to be present in the plasma membranes of both central and peripheral cells and participate in detecting temperature changes (Wang and Siemens, 2015; Kashio, 2021), thus playing a critical role in the entrainment of peripheral clocks (Bromberg et al., 2013; Poletini et al., 2015). Temperature is one of the gating forces to the thermo-TRPs activation (Matta and Ahern, 2007; Yao et al., 2011; Xiao and Xu, 2021), which means that they can be fully activated only by temperature changes that either fall within a specific temperature range or exceed a particular temperature threshold. This implies that both magnitude and changes in temperature are detected. Such temperature sensing process is expected to be essential for temperature entrainment and is assumed to be the first step of the system’s response to a temperature input. Using an indirect response modeling (IRM) approach (Foteinou et al., 2009; Foteinou et al., 2011), we hypothesize two intermediate signals, denoted as *TS*_*1*_ and *TS*_*2*_, to represent the essential components involved in this cascade. Eqs 1, 2 describe their kinetics.

By using the average to represent, for simplicity, the magnitude of the input temperature oscillation, Eq. 2 encapsulates the temperature-induced thermo-TRPs activation upon detecting both temperature magnitude and change as well as their inactivation as a result of the homeostatic regulation. This transient activation of thermo-TRPs in the membrane in response to the temperature rhythm might enable the influx of cation ions and the change of membrane fluidity, which then initiate other downstream intracellular events (Vriens et al., 2014; Wang and Siemens, 2015; Guihur et al., 2022). Eq. 3 generalizes the surge (denoted by a Hill coefficient) and the vanishment of the effector of the thermosensor’s activation.

HSF1 is hypothesized to be involved in the mammalian temperature entrainment through the HSF1-induced heat shock response (HSR) pathway (Buhr et al., 2010). When there is no stress in the canonical HSR pathway, heat shock proteins (HSPs) sequester HSF1 monomers and suppress HSF1 transcriptional activity. Upon heat stress, the denaturation of specific thermolabile proteins leads to the dissociation of HSP chaperones from inactive HSF1/HSP complexes. Then, hyperphosphorylation, trimerization, and translocation of HSF1 occur sequentially (Rieger et al., 2005; Reinke et al., 2008; Scheff et al., 2015; Mazaira et al., 2018; Dudziuk et al., 2019; Masser et al., 2020; Guihur et al., 2022), inducing transcriptional activity of HSF1. The active HSF1 trans-activators stimulate the production of HSPs by binding the heat shock elements (HSEs) on the promotor. The resulting excessive HSPs in turn engage in the termination of HSF1 activation with the same sequestration strategy (Gomez-Pastor et al., 2018; Dudziuk et al., 2019). Independent of this molecular chaperone displacement theory, HSF1 can also be activated through different mechanisms, such as temperature-induced intrinsic structural response and other inter-molecular interactions (Hentze et al., 2016; Gomez-Pastor et al., 2018; Mazaira et al., 2018). However, exactly how HSF1 is activated by temperature variations within the physiological range and how HSR participates in the entrainment process remains unclear.

Therefore, by considering the contribution of thermosensors to thermoregulation and focusing on the HSF1-induced excessive HSPs that play an essential role in recovering HSF1 to its inactivated state, we assume that the HSF1 responds to a temperature input (*TS*_*2*_) as is transduced by the thermosensing process while being inhibited by the excessive HSPs. The mechanisms are expressed via Eq. 4, which delineates the transduction of the temperature signal leading to the HSPs-mediated activation and the homeostatic inactivation of HSF1. Besides this canonical pathway, HSPs are also found to promote the noncanonical dissociation of active trimer HSF1 from the HSEs on target DNA (Kijima et al., 2018). This is described by HSPs inhibiting their HSF1-induced production in Eq. 5 along with a 1^st^-order protein degradation of HSPs.

Moreover, studies suggest that the HSF1 activity, such as its nuclear level, phosphorylation degree, and HSE-binding, exhibits circadian rhythm (Reinke et al., 2008; Hirota et al., 2016). In contrast, the *Hsf1* mRNA and total HSF1 protein levels remain constant throughout the day (Buhr et al., 2010; Hirota et al., 2016). As such, the transcription dynamics of *Hsf1* mRNA are not included.

$$temperature\;\left(T,\mathrm{{}^{\circ}C}\right)=\left\{\begin{array}{l} 37+\frac{∆T}{2},\;0\leq ZT<12\\ 37-\frac{∆T}{2},\;12\leq ZT<24 \end{array}\right.$$ (1)

$$\frac{d{TS}_{1}}{dt}=v_{act0,ts1}\;T_{avg}+v_{act1,ts1}\left(T-T_{avg}\right)-v_{ina,ts1}{\;TS}_{1}$$ (2)

$$\frac{d{TS}_{2}}{dt}=\frac{v_{b,ts2}\;{{TS}_{1}}^{n}}{{k_{b,ts2}}^{n}+{{TS}_{1}}^{n}}-v_{d,ts2}{\;TS}_{2}$$ (3)

$$\frac{dactHSF1}{dt}=v_{act,hsf1}\frac{\left({HSF1}_{tot}-actHSF1\right)}{indHSP}\left[1+\frac{k_{T}\;{TS}_{2}}{K_{T}+{TS}_{2}}\right]-v_{ina,hsf1}actHSF1$$ (4)

$$\frac{dindHSP}{dt}=v_{b,hsp}actHSF1/indHSP\;-v_{d,hsp}indHSP$$ (5)

The active HSF1 transduces the circadian oscillations of the temperature signal to the peripheral clock genes by inducing *Per2* transcription (Saini et al., 2012). In addition, an active HSF1:CLOCK/BMAL1 interaction on the *Per2* promotor was observed after a heat shock pulse and speculated to be involved in the temperature resetting process (Tamaru et al., 2011). Thus, This interaction is hypothesized to stabilize the HSF1:HSE binding and be competitive with the HSP noncanonical function. These mechanisms are included in the HSF1 entraining term in Eq. 6, which models the induction effect of the active HSF1 trans-activator on the *Per/Cry* expression as an indirect response.

### Peripheral clock gene dynamics

The intrinsic dynamics of the peripheral clock gene network (Eqs 6–12) are modeled based on previous works (Becker-Weimann et al., 2004; Geier et al., 2005; Mavroudis et al., 2012; Mavroudis et al., 2014; Li and Androulakis, 2021). The network consists of transcriptional translational feedback loops, incorporating intertwined positive and negative feedback loops (Becker-Weimann et al., 2004; Geier et al., 2005). Through a positive feedback loop, the nuclear PER/CRY protein indirectly activates the transcription of *Bmal1* mRNA. The translation to cytoplasmic BMAL1 protein and its translocation to the nucleus (nucBMAL1) lead to an increase in the production of CLOCK/BMAL1 heterodimer. On the other hand, the nuclear PER/CRY inhibits the stimulation of the transcription of *Per/Cry* by CLOCK/BMAL1.

$$\frac{d{Per/Cry}_{mRNA}}{dt}=\frac{v_{1b}\;\left(CLOCK/BMAL1+c\right)}{k_{1b}\;\left(1+{\left(\frac{nucPER/CRY}{k_{1i}}\right)}^{p}+CLOCK/BMAL1+c\right)}\;\left[1+\frac{k_{hsf1}\;actHSF1}{K_{hsf1}+actHSF1+k_{hsf1,ci}\frac{indHSP}{CLOCK/BMAL1}}\right]-k_{1d}\;{Per/Cry}_{mRNA}$$ (6)

$$\frac{dPER/CRY}{dt}=k_{2b}\;{{Per/Cry}_{mRNA}}^{q}-k_{2d}\;PER/CRY-k_{2t}\;PER/CRY+k_{3t}\;nucPER/CRY$$ (7)

$$\frac{dnucPER/CRY}{dt}=k_{2t}PER/CRY-k_{3t}\;nucPER/CRY-k_{3d}\;nucPER/CRY$$ (8)

$$\frac{d{Bmal1}_{mRNA}}{dt}=\frac{v_{4b}\;{nucPER/CRY}^{r}}{{k_{4b}}^{r}+{nucPER/CRY}^{r}}-k_{4d}\;{Bmal1}_{mRNA}$$ (9)

$$\frac{dBMAL1}{dt}=k_{5b}\;{Bmal1}_{mRNA}-k_{5d}\;BMAL1-k_{5t}\;BMAL1+k_{6t}\;nucBMAL1$$ (10)

$$\frac{dnucBMAL1}{dt}=k_{5t}\;BMAL1-k_{6t}\;nucBMAL1-k_{6d}\;nucBMAL1+k_{7a}\;CLOCK/BMAL1-k_{6a}\;nucBMAL1$$ (11)

$$\frac{dCLOCK/BMAL1}{dt}=k_{6a}\;nucBMAL1-k_{7a}\;CLOCK/BMAL1-k_{7d}\;CLOCK/BMAL1$$ (12)

### Parameter estimation

Fifteen newly introduced parameters associated with the temperature signaling cascade and entrainment process were estimated, while the remaining parameters were set to the values determined earlier (Mavroudis et al., 2012). The parameter estimation aimed to satisfy the following criteria at the single-cell level: 1) the concentrations of *TS*_*1*_, *TS*_*2*,_ and activated HSF1 remain positive; 2) when subject to a 12-h warm/12-h cold (W12/C12 37±1.5 ℃ ) temperature oscillation, the clock genes must be entrained to a 24-h period; 3) the phase of cytoplasmic PER/CRY fits experimental data (Saini et al., 2012). In this study, the phase of a component is defined as the periodically stable difference between its peaking time and the onset of the warm phase of the temperature cycle (ZT0). The period is calculated by averaging the time differences between successive peaks during the component’s steady oscillation stage. The estimation also accounts for the fact that the TS signaling cascade would leamplify of the signal and, furthermore, that the level of (activation of) HSF1 is likely similar to that of the PCGs (Kaneko et al., 2020). The nominal parameter values of the model are summarized in Table 1.

**TABLE 1:** Model parameters and their nominal values.

#	Parameters	Values	Units	Descriptions	Sources
1	$v_{act0,ts1}$	0.13	$nM∙h^{-1}∙{℃}^{-1}$	Activation rate of *TS*_*1*_ (thermosensors) in response to the magnitude (average) of the temperature rhythm	Estimated
2	$v_{act1,ts1}$	0.65	$nM∙h^{-1}∙{℃}^{-1}$	Activation rate of *TS*_*1*_ in response to the variation (amplitude) of the temperature rhythm	
3	$v_{ina,ts1}$	0.65	$h^{-1}$	Inactivation rate of *TS*_*1*_	
4	$v_{b,ts2}$	8	$nM∙h^{-1}$	Maximal rate of *TS*_*2*_ (effector of thermosensors activation) production	
5	$k_{b,ts2}$	20	nM	Michaelis constant of *TS*_*2*_ production	
6	n	3	1	Hill coefficient of activation of *TS*_*2*_ production	
7	$v_{d,ts2}$	0.45	$h^{-1}$	Degradation rate of *TS*_*2*_	
8	$k_{T}$	27.17	1	Effect strength of transduced temperature input (*TS*_*2*_)	
9	$K_{T}$	3.68	nM	Michaelis constant of transduced temperature input (*TS*_*2*_)	
10	$v_{act,hsf1}$	0.33	$nM∙h^{-1}$	Activation rate of HSF1	
11	${HSF1}_{tot}$	20.89	nM	Total HSF1 concentration	
12	$v_{ina,hsf1}$	20.65	$h^{-1}$	Inactivation rate of HSF1	
13	$v_{b,hsp}$	8.72	$nM∙h^{-1}$	Production rate of active HSF1-induced HSP	
14	$v_{d,hsp}$	2.37	$h^{-1}$	Degradation rate of active HSF1-induced HSP	
15	$k_{hsf1}$	39.97	1	Coupling strength of active HSF1 on *Per/Cry* transcription	
16	$K_{hsf1}$	1.07	nM	Michaelis constant of stimulation of *Per/Cry* transcription by active HSF1	
17	$k_{hsf1,ci}$	1	nM	Competency of inhibitory effect of active HSF1-induced HSP with respect to stabilized effect of CLOCK/BMAL1	
18	$v_{1b}$	9	$nM∙h^{-1}$	Maximal rate of *Per/Cry* transcription	Pierre et al. (2016)
19	c	0.01	nM	Concentration of constitutive activator	
20	$k_{1b}$	1	nM	Michaelis constant of *Per/Cry* transcription	
21	$k_{1i}$	0.56	nM	Inhibition constant of *Per/Cry* transcription	
22	p	8	1	Hill coefficient of inhibition of *Per/Cry* transcription	
23	$k_{1d}$	0.12	$h^{-1}$	Degradation rate of *Per/Cry* mRNA	
24	$k_{2b}$	0.3	${nM}^{-1}∙h^{-1}$	Complex formation rate of *Per/Cry* mRNA	
25	q	2	1	Number of PER/CRY complex forming subunits	
26	$k_{2d}$	0.05	$h^{-1}$	Degradation rate of cytoplasmatic PER/CRY	
27	$k_{2t}$	0.24	$h^{-1}$	Nuclear import rate of the PER/CRY complex	
28	$k_{3t}$	0.02	$h^{-1}$	Nuclear export rate of the PER/CRY complex	
29	$k_{3d}$	0.12	$h^{-1}$	Degradation rate of the nuclear PER/CRY complex	
30	$v_{4b}$	3.6	$nM∙h^{-1}$	Maximal rate of *Bmal1* transcription	
31	$k_{4b}$	2.16	nM	Michaelis constant of *Bmal1* transcription	
32	r	3	1	Hill coefficient of activation of *Bmal1* transcription	
33	$k_{4d}$	0.75	$h^{-1}$	Degradation rate of *Bmal1* mRNA	
34	$k_{5b}$	0.24	$h^{-1}$	Translation rate of BMAL1	
35	$k_{5d}$	0.06	$h^{-1}$	Degradation rate of cytoplasmatic BMAL1	
36	$k_{5t}$	0.45	$h^{-1}$	Nuclear import rate of BMAL1	
37	$k_{6t}$	0.06	$h^{-1}$	Nuclear export rate of BMAL1	
38	$k_{6d}$	0.12	$h^{-1}$	Degradation rate of nuclear BMAL1	
39	$k_{6a}$	0.09	$h^{-1}$	Activation rate of the nuclear CLOCK/BMAL1	
40	$k_{7a}$	0.003	$h^{-1}$	Deactivation rate of the CLOCK/BMAL1 complex	
41	$k_{7d}$	0.09	$h^{-1}$	Degradation rate of the CLOCK/BMAL1 complex	

### In silico population and quantification of synchronicity

A population of cells and individuals is simulated in the *in silico* experiments in this study. The cell population is used to understand the ensemble (average) behavior of peripheral cell oscillators, representative of tissue behavior (Yamazaki et al., 2002; Liu et al., 2007; Abraham et al., 2010), while members of the ensemble of individuals are utilized to approximate personalized responses (intra- and inter-individual variability). We generate 1,000 cells for the cell population by sampling the parameters associated with the temperature signaling cascade and peripheral clock gene network. To simulate inter-individual variability, we assume subjects to have individualized temperature sensing ability, which is attained by sampling the associated parameters ( $v_{act0,ts1}$ , $v_{act1,ts1}$ and $k_{b,ts2}$ ). Sampling is accomplished using the Sobol method (Rao and Androulakis, 2017; Scherholz et al., 2020).

To explore how the circadian rhythm of the temperature signal affects the state of clock genes at the peripheral tissue level, we evaluate the synchronicity of the cell population. In particular, the synchronization, defined as *R*_*syn,j*_, is assessed by quantifying cell oscillators’ deviations from mean levels (in terms of the clock gene component j ) and is calculated by dividing the variance of the mean field by the variance of each oscillator (Mavroudis et al., 2012).

$$R_{syn,j}=\frac{\langle {\bar{y}}_{j}^{2}\rangle -{\langle {\bar{y}}_{j}\rangle }^{2}}{\frac{1}{N}\sum_{i=1}^{N}\left(\langle y_{j,i}^{2}\rangle -{\langle y_{j,i}\rangle }^{2}\right)}$$ (13)

where

$${\bar{y}}_{j}=\frac{1}{N}\sum_{i=1}^{N}y_{j,i}$$ (14)

$$\langle {\bar{y}}_{j}\rangle =\frac{1}{t_{T}}\sum_{t=t_{1}}^{t_{T}}{\bar{y}}_{j}\left(t\right)$$ (15)

In Eq. 13, $y_{j,i}$ is the time-course vector output generated by the model equations, where the indexes j and i represent the component and the cell (*N* total), respectively. ${\bar{y}}_{j}$ is the time-course vector of averages over the population of *N* cells (Eq. 14) and $\langle ∎\rangle$ is the time average (timespan from $t_{1}$ to $t_{T}$ ) (Eq. 15). A minimum value of 0 of *R*_*syn,j*_ indicates an entirely desynchronized state and a maximum value of 1 demonstrates a full synchronization. In this study, *R*_*syn,j*_ is calculated for the *actHSF1* and/or *Per/Cry* mRNA components for a timespan of 1200 h, except for the validation and circadian disruption experiments in which it is continuously calculated for every 720 h and 24 h, respectively.

### Modeling “temperature shift”

The circadian patterns of core body temperature are controlled by the mammalian master clock in the SCN, which is entrained by light/dark cycles (Edery, 2010; Morf and Schibler, 2013; Coiffard et al., 2021). Changes in the phase and period of the light/dark cycles may affect the circadian body temperature rhythm’s phase, period, and amplitude. This is manifested clearly in shift work, a type of circadian disruption, and has been recorded in many *in vivo* experiments (Knauth et al., 1978; Reinberg et al., 1980; Knauth et al., 1981; Reinberg et al., 1988; Logan and McClung, 2019). Since such mechanisms are absent in cell cultures, we hypothesize that imposed temperature rhythms can play the same *zeitgeber* role in cultured cells, offering the opportunity to mimic circadian disruption *in vitro* (Abraham et al., 2010; Stowie et al., 2019).

By applying persistent perturbations to the imposed temperature rhythm, permanent or alternating “temperature shift” are simulated in our study to examine the long-term effect of circadian disruption on peripheral cell synchronization and the inter-individual variability of responses to circadian disruption. As mentioned earlier, the temperature pattern is simulated as a series of Heaviside step functions (Eq. 1). In other words, during the warm period, the temperature is set at its maximum, whereas during the cold period, the temperature is set at its minimum. Combining different patterns results in various *zeitgeber* schedules. Thus, an alternating shift schedule (ASS) consists of a combination of successive inverted patterns (hereinafter termed “normal” and “reversed”, respectively):

$$normal\;T\;\left(T_{n}\right)=\left\{\begin{array}{c} T_{\max},\;0\leq ZT<12\\ \;T_{\min},\;12\leq ZT<24 \end{array}\right.$$ (16)

$$reversed\;T\;\left(T_{r}\right)=\left\{\begin{array}{c} T_{\min},\;0\leq ZT<12\\ \;T_{\max},\;12\leq ZT<24 \end{array}\right.$$ (17)

**FIGURE A1:**
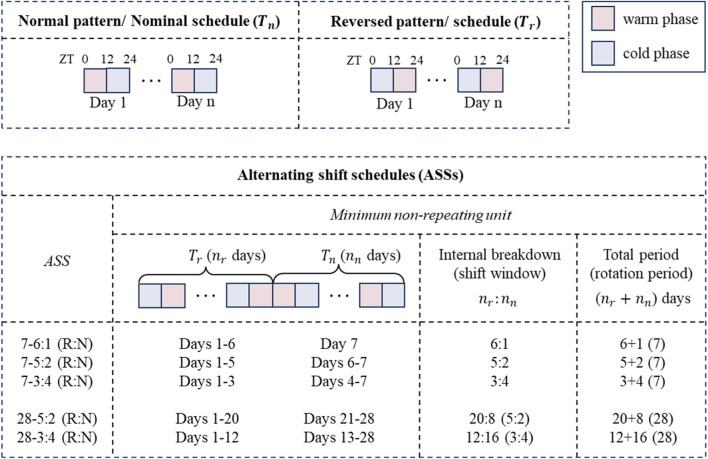
Schematic of temperature schedules. The nominal temperature schedule comprises recurring normal temperature patterns ( $T_{n}$ ). The alternating shift schedules (ASSs) consist of the periodic alternation between $n_{r}$ -day normal temperature patterns ( $T_{n}$ ) and $n_{n}$ -day reversed patterns ( $T_{r}$ ). The ratio $n_{r}:n_{n}$ within each minimum non-repeating unit of an ASS defines the schedule’s internal breakdown (or shift window), while the sum $n_{r}+n_{n}$ , the total period of the minimum non-repeating unit, defines the schedule’s rotation period.

The following temperature schedules are examined: 1) 5-day reversed (R) and 2-day normal (N) shift, 2) 3-day reversed and 4-day normal shift, 3) 6-day reversed and 1-day normal shift every 7 days (essentially simulating, in shorthand, 7–5:2/−3:4/−6:1 (R:N) ASS); 4) 20-day reversed, and 8-day normal shift, 5) 12-day reversed and 16-day normal shift every 28 days (in shorthand, a 28–5:2/−3:4 (R:N) ASS). Schedules (i)-(iii) and (iv)-(v) hold the same rotation periods, respectively, whereas (i) and (iv), (ii), and (v) have the same shift windows, respectively. A detailed depiction of $T_{n}$ , $T_{r}$ , and ASSs is provided in Figure A1.

The modeling and computational analyses were implemented in MATLAB R2020b, and the codes are available in GitHub (https://github.com/IPAndroulakis/Temperature-induced-CR).

## Results

### Model calibration and validation

To simulate the peripheral entrainment subject to an imposed temperature rhythm, we constructed the model with two parts: (a) temperature signaling cascade and (b) peripheral clock gene network (Figure 1; see details in Materials and Methods). In the temperature signaling cascade, we consider a generalized thermosensor-transductor dynamic model and the downstream effector represented by an HSF1-mediated HSR pathway. The peripheral clock gene network is built upon a gene regulatory network model (Jong, 2002) as our earlier works (Mavroudis et al., 2012; Mavroudis et al., 2014; Mavroudis et al., 2015), while temperature rhythms are modeled as earlier described, with a 12 h warm (inactive) period between *zeitgeber* time (ZT) 0 h–12 h and the cold (active) period between ZT 12 h–24 h (nocturnal animals).

**FIGURE 2:**
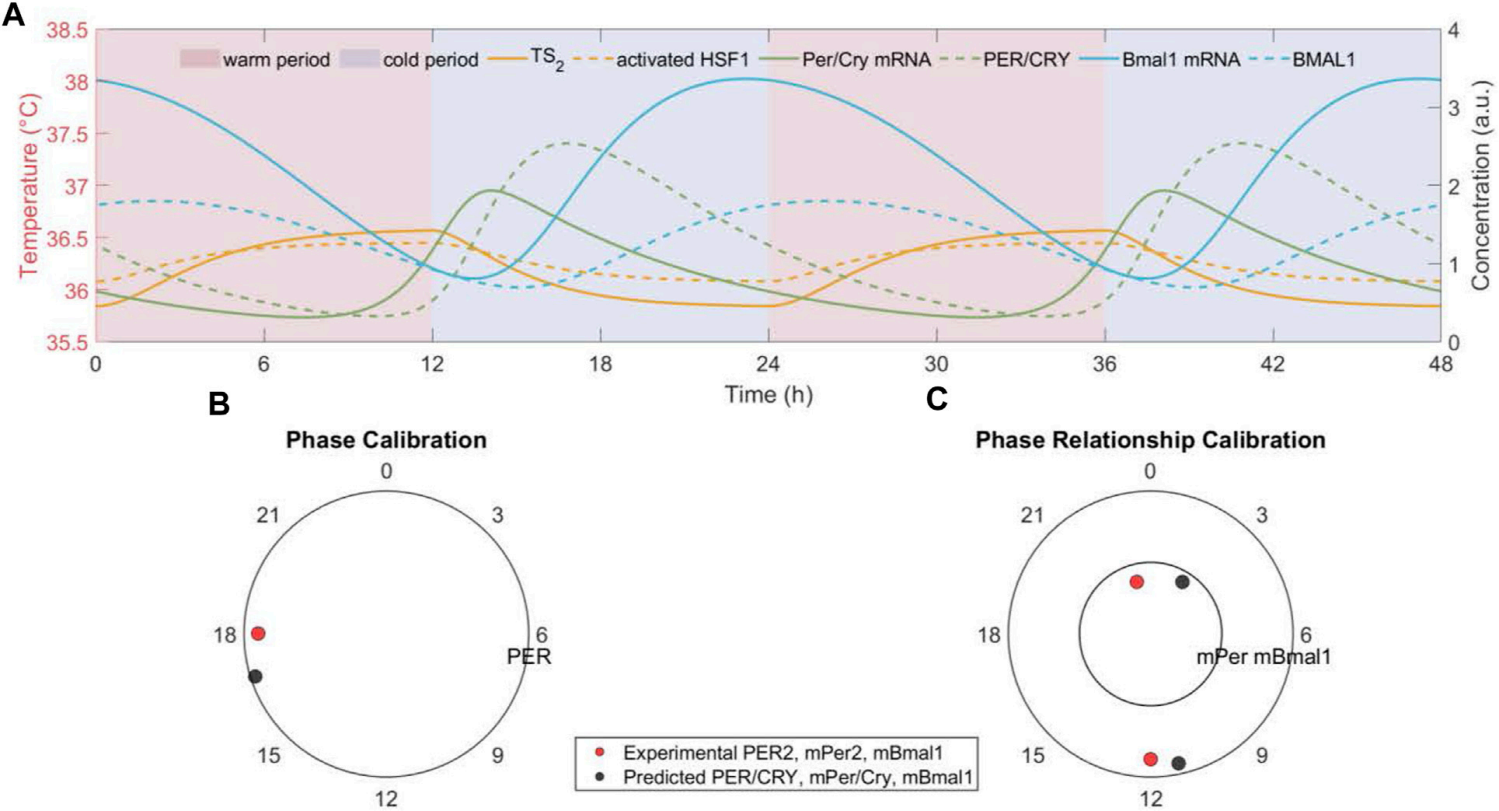
The peripheral cell dock gets phase and period entrained to the imposed temperature rhythm. **(A)** The temporal dynamics of transduced temperature signal (*TS_2_*), activate HSF1, clock genes (*Per/Cry* and *Bmal1*) mRNAs and cytoplasmic proteins, **(B)** the phase of clock gene (*Per/Cry*) cytoplasmatic protein, and **(C)** the phase relationship between clock genes (*Per/Cry* and *Bmal1*) mRNAs generated by the model with nominal parameters indicated in Table 1 and subject to a nominal W12/C12 37°C ± 1.5°C temperature rhythm. All components oscillate with a period of 24 h and maintain a stable phase relationship with the onset of the cold phase in the temperature rhythm. In addition, the predicted *Per/Cry* phase and nearly antiphase relationship between *Per/Cry* and *Bmal1* mRNAs are experimentally consistent (Becker-Weimann et al., 2004; Li and Androulakis, 2021). Red/blue patterns denote warm/cold settings.

We hypothesize that temperature rhythms drive the phase and period of the peripheral cell-autonomous oscillators. Consequently, all the clock components’ rhythms are expected to be entrained and maintain stable temperature-phase relations (Abraham et al., 2018). Figure 2A reflects the temporal dynamics of representative components (temperature signal (*TS*_*2*_), HSF1 activity, clock genes’ mRNA, and their cytoplasmic proteins) when cells are entrained to a nominal W12/C12 37±1.5 ℃ temperature rhythm. Figures 2B,C show that the model reproduces the cytoplasmic PER/CRY protein phase and the antiphase relationship between *Per/Cry* and *Bmal1* mRNA as observed experimentally (Brown et al., 2002; Saini et al., 2012).

**FIGURE 3:**
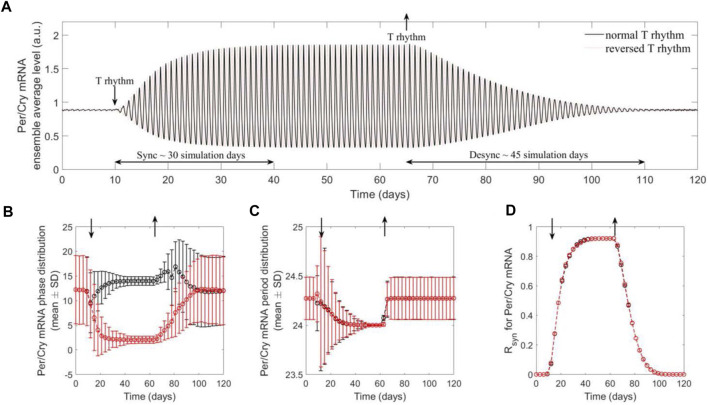
The population of peripheral cell clocks gets entrained and reset to the present rhythms of temperature signals. The population behavior was characterized by the ensemble average of cell clocks. Two temperature schedules were imposed, which are comprised of a consecutive absence (constant temperature at 37°C) and presence (W12/C12 37°C ± 1.5°C with 12 h phase difference) of the rhythms. The *Per/Cry* mRNA ensemble average circadian profile **(A)**, single-cell phase **(B)** and period **(C)** distributions, and synchrony degree (*R_syn_*) dynamics **(D)** were generated to analyze synchronization and desynchronization properties of component cell clocks. By preventing cell clocks from maintaining their original intrinsic/incoherent phases and periods, the presence of rhythm drives the synchronization of these individual oscillators, as indicated by shifted, narrowly distributed phases and periods, and high synchrony. This leads to the enhanced ensemble average amplitude and the adaptation of ensemble average phase and period to temperature rhythms. These results demonstrate efficient entraining and resetting effects of temperature rhythm on a population of cells. In addition, the difference in the dynamics of synchronization and desynchronization may be attributed to the inference of external signals in the intrinsic interactions between clock genes with interlocking feedback loops.

Our model further captures the entraining and resetting effect of temperature rhythm on the synchronization of a population of cells. Figure 3A depicts the ensemble dynamics of *Per/Cry* mRNA of a cell population subject to a switch between introduction and removal of temperature rhythm. In the absence of temperature rhythm $\left(t<10\;d\right)$ , the system experiences a constant temperature T=37 ℃ . The system is then exposed to temperature oscillations and returns to the isothermal condition at t=65 d . We observe the onset and stabilization of robust oscillations induced by temperature rhythms, which dampen once the temperature is fixed again. The system was entrained to both normal and reversed temperature profiles (Eq. 16). Depending on the temperature profile, the ensembles are entrained to the appropriate phases (Figure 3B), whereas in both cases, the ensembles are entrained to the 24-h temperature period (Figure 3C). Finally, once the temperature rhythms are imposed, the system reaches a high level of synchronization (Figure 3D), and once the *zeitgeber* is removed, the ensemble of peripheral cells quickly relaxes to the dispersed intrinsic phase and period distributions.

Interestingly, the qualitative differences in the dynamics of synchronization and desynchronization are apparent: the system requires a substantially shorter time to synchronize than desynchronize. This model prediction can be explained by *Per2*’s property of immediate early gene (Saini et al., 2012), suggesting that during the period following receiving an input signal, *Per/Cry* in each cell oscillator functions as an immediate early regulator. However, once the driving force is removed, *Per/Cry* functions solely as a core clock gene, leading the network to relax more gradually via its intrinsic mutual interactions between components and interlocking feedback loops.

### The effects of temperature characteristics on peripheral clock synchronization

**FIGURE 4:**
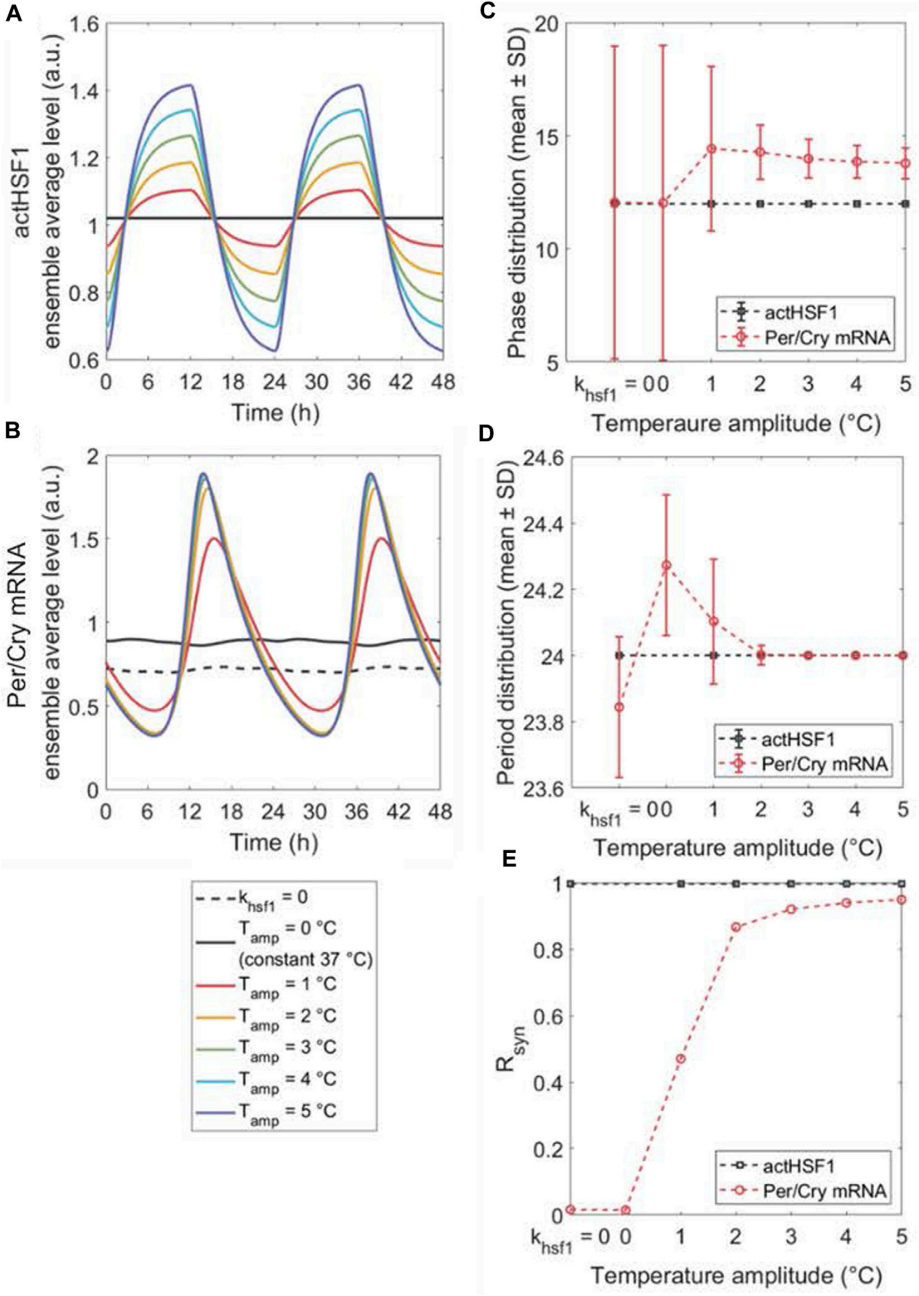
The peripheral synchronization changes in response to amplitude-varying temperature rhythms. The dynamics of ensemble average **(A,B)**, single-cell phase **(C)** and period **(D)** distributions, and synchronization degree *R_syn_* **(E)** of *actHSF1* and *Per/Cry* mRNA were obtained when peripheral cells are either insusceptible to (by setting *k_hsf1_* = 0 in the model) or susceptible to temperature rhythms with amplitudes varying from 0°C to 5°C. The loss of entraining agent (*k_hsf1_* = 0) and the elimination of the entrainer rhythm (constant temperature) result in a similar response of cell population, with the only difference in the magnitude of *Per/Cry* mRNA ensemble average and period. The increase in the amplitude of temperature rhythm enhances the *Per/Cry* mRNA ensemble average amplitude by more efficiently synchronizing the peripheral cells. This enhancement relies on both the amplification of rhythmic *actHSF1* inputs and the inherent oscillating property of clocks.

Since *zeitgeber*’s strength is critical for entrainment and synchronization, we evaluated the synchronization response to variations in the temperature amplitude. By varying the peak-to-trough values, i.e., amplitude ( ΔT=1−5 ℃ ), we simulated the increase in *zeitgeber*’s strength, which leads to an amplification of the actHSF1 ensemble average (Figure 4A) and consequently the *Per/Cry* mRNA ensemble average (Figure 4B). However, the former is primarily the result of the temperature-induced amplitude amplification of the single-cell actHSF1 oscillation, whereas the latter results from the increased synchronization of cells leading to robust *Per/Cry* mRNA oscillations. The increase in ensemble average amplitude as a result of entraining the cells is also indicated by the tightening of phase and period distribution (Figures 4C,D). Finally, Figure 4E quantifies the enhancement in peripheral synchronization as a function of the temperature oscillation amplitude. Our prediction that HSF1 activity does not exhibit a circadian rhythm when there is no fluctuation in temperature are consistent with experimental evidence suggesting that HSEs are not transactivated at a constant temperature (Reinke et al., 2008; Tamaru et al., 2011). The model also captures the observations (Refinetti, 2020) that low-amplitude temperature rhythm can partially synchronize the cells (Refinetti, 2020), and cellular oscillators are synchronized more efficiently with increasing temperature amplitudes. However, the complete elimination of temperature oscillations is approximately equivalent to a loss of the entraining signal, as confirmed by the prediction for $k_{hsf1}=0$ . Furthermore, our results in Figure 4 indicate that an inherent/intrinsic robust oscillation enables the peripheral clock gene network to be capable of gradually/progressively (with more resistance) adapting to external rhythmic forces. In contrast, the temperature signaling cascade, lacking its own inherent rhythm in the absence of input signals, exhibits an immediate and facile adaptation to the same external forces. This difference can also be observed in Figures 4C–E that the *actHSF1*’s period and phase adapt with high synchrony to all temperature rhythms with varying amplitudes, which is denoted by the narrowly distributed individual phases and periods and the synchronization index ( $R_{syn}$ ) of 1.

Similarly, by altering the *zeitgeber*’s average ( 32−40 ℃ with fixed amplitude ΔT=3 ℃ ), which is rationalized by the experimental evidence showing the core body temperature in humans can maintain its circadian rhythm even in plasmodium parasite-induced fever (Lell et al., 2000), we found that increasing the average of temperature enhanced the average of the single-cell actHSF1 oscillation and thus the actHSF1 ensemble average oscillation (Supplementary Figure S1A). However, this did not translate to an apparent change in peripheral synchronization (Supplementary Figure S1B), which is consistent with experimental findings suggesting that elevated temperatures do not lead to loss of peripheral circadian rhythms and only slightly impact the rhythms’ current characteristics (Saini et al., 2012). Combined with the previous results, it indicates that the variation (amplitude), as opposed to the magnitude (average) of temperature oscillations, is more critical for the phase and period entrainment and the synchronization of peripheral cellular oscillators (Saini et al., 2012).

To further elucidate how the period of temperature oscillations influences the synchronization of peripheral clocks, we analyzed the effect of period mismatch between the ensemble average of peripheral cell oscillators (intrinsic period ∼ 23.8 h ) and *zeitgeber* on ensemble entrainment. Following the approach proposed by Schmal et al., 2015, we characterized the ensemble synchronization level and average entrainment phase on the *zeitgeber* period - *zeitgeber* amplitude parameter plane (Supplementary Figure S2). As expected, the *Arnold tongue* indicates that period mismatch leads to desynchronization that can be partially overcome by increasing temperature amplitude; in turn, decreasing period mismatch can compensate for the weak synchronization power of lower temperature amplitudes (Supplementary Figure S2A). These are also reflected by the peripheral clocks being able to adapt to a broader range of period length when subjected to increasing amplitudes of temperature rhythms, along with more pronounced divergences in the ensemble average entrainment phase and its contrast with the *zeitgeber*’s phase (defined as the onset of the cold period) (Supplementary Figures S2B, C). In particular, our results suggest that the ensemble average entrainment phase is exquisitely sensitive to temperature rhythms, as evidenced by a phase advance (relative to temperature oscillation) in cells entrained to shorter periods (Supplementary Figure S2C). This finding may reflect the concept of overcompensation and is consistent with the experimental result that a modest change in *zeitgeber*’s period can trigger a significant phase shift, leading to a substantial difference between the phases of the external time cue and internal clock (Duffy et al., 2001).

### Internal responses to alternating temperature schedules

The previous results established relations between the synchronization behavior and dynamics of peripheral clocks and unperturbed temperature oscillations. In this subsection, we will investigate and analyze the response of a system exposed to alternating temperature schedules (i.e., perturbed temperature oscillations), which we describe as “alternating shift schedules (ASSs).”

**FIGURE 5:**
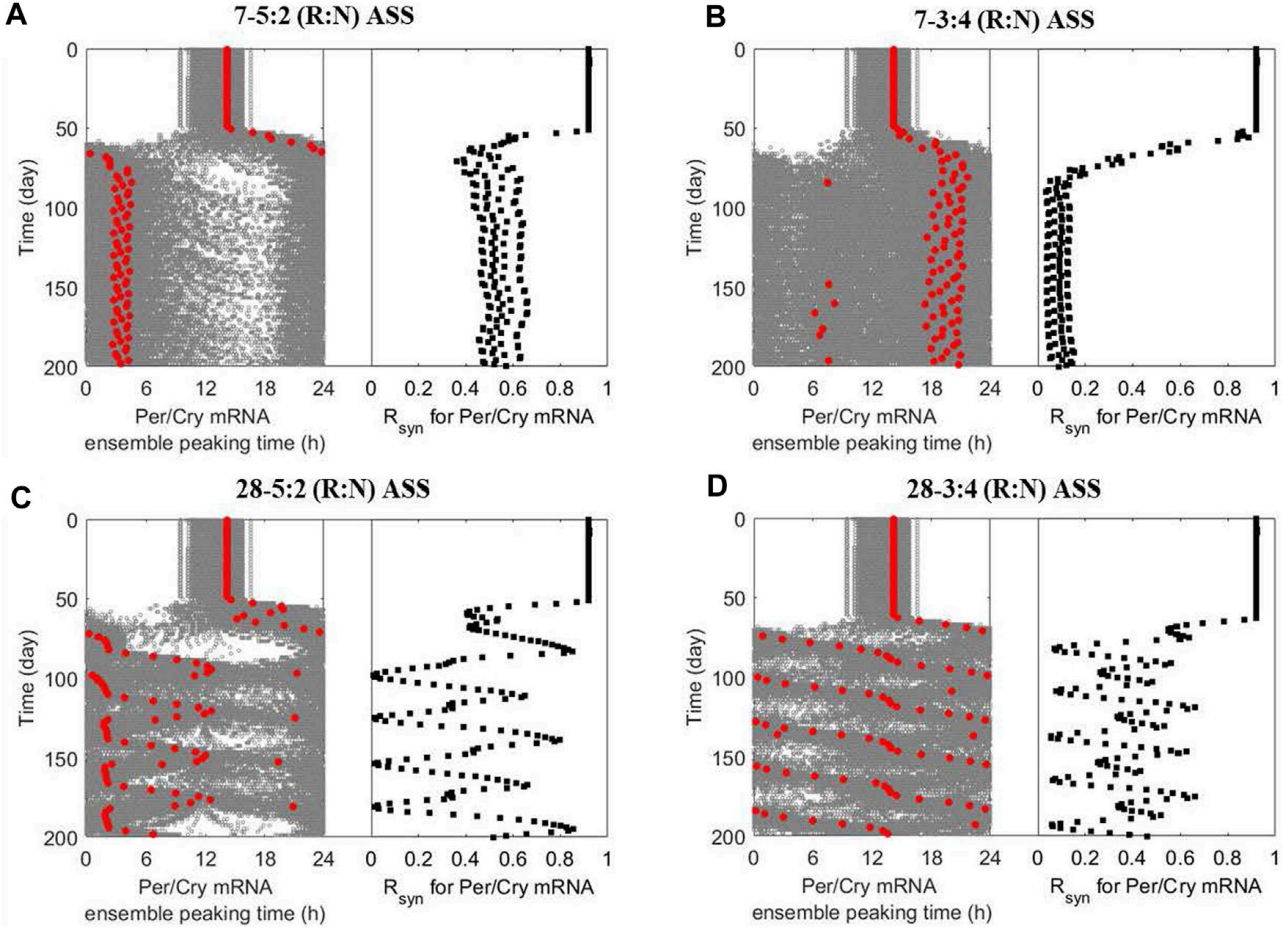
The multicellular system responds to alternating shift schedules (ASSs) in a schedule-dependent manner. The dynamics of ensemble phases (left panel; single-cell phases and their ensemble average denoted by grey and red circles, respectively) and synchronization level (right panel) of peripheral cell clocks were generated when the multicellular system was subject to different ASSs after 50 simulation days: **(A)** 5-day reversed and 2-day normal (7–5:2 (R:N)), **(B)** 3-day reversed and 4-day normal (7–3:4 (R:N)), **(C)** 20-day reversed and 8-day normal (28–5:2 (R:N)) and **(D)** 12-day reversed and 16-day normal (28–3:4 (R:N)). The system exhibits better circadian adaptation under the 7–5:2 (R:N) than the 7–3:4 (R:N) ASS, as indicated by eventually adopting oscillatory properties (phase and period) of the dominating temperature pattern and maintaining a higher internal synchronization. The slowly rotating schedules (28–5:2 and −3:4 (R:N) ASSs) generally damage the system’s circadian adaptive ability by inducing severe fluctuation in its dynamic phase entrainment and synchronization, implying an underlying destabilization compared to frequently rotating schedules (7–5:2 and - 3:4 (R:N) ASSs).

Our first *in silico* experiments mimic the equivalence to “shift work,” where the system is repeatedly exposed to one z*eitgeber* schedule followed by a different schedule, each lasting for a certain period of time. Figure 5 shows the dynamics of ensemble phases and synchronization level of peripheral clocks subject to different ASSs (Figure A1). Two parameters define the ASS: a) the total period of the minimum non-repeating unit of schedule and b) the internal breakdown. For example, “7–5:2” implies that we consider a 7-day period during which the system is exposed to one oscillatory pattern for 5 days, and then the pattern is inverted for 2 days. Then, the 7-day cycle repeats in the schedule. Similarly, “7–4:3” implies a 7-day period with 4 days in one pattern and 3 days in the inverted. On the other hand, “28–5:2” means that we consider a 28-day period during which the system is exposed to one temperature pattern for 20 days and then an inverted pattern for 8 days (5:2 is the ratio of days in each pattern). Equivalently, “28–3:4” implies a 28-day non-repeating unit, with 12 days in one pattern and 16 days in the inverted. Specifically, in the simulations that follow, the system is first exposed to a “normal” temperature pattern ( $T_{n}$ , Eq. 16) and then subjected to a schedule that alternates between $T_{n}\text{ and }T_{r}$ (Eq. 17) at t=50d .

Figure 5A indicates that the ensemble average of peripheral clocks in a system following a 7–5:2 (R:N) schedule eventually adopts oscillatory characteristics resembling the dominating temperature pattern with a phase advance of −12 h while maintaining robust synchronization in the long run. The manifestation is quite different under the 7–4:3 (R:N) schedule (Figure 5B), where the system never manages to attain a clear phase and achieve synchronization. Thus, this schedule induces significant desynchrony. Circadian misalignment and desynchronization of cellular clocks are major contributing factors to chronic disease prevalence. Our observations are consistent with the experimental finding showing the three-shift workers are more vulnerable (probably by having a more dysregulated immune system (Castanon-Cervantes et al., 2010)) than the five-shift workers, resulting in a higher prevalence of common infections among members of the first category (Mohren et al., 2002).

We then considered slowly rotating schedules (i.e., longer time on each pattern before a change happens), generating more extended periods for the minimum non-repeating units. The peripheral ensemble average phase (*Per/Cry* mRNA peaking time) fluctuates significantly upon exposure to a 28–5:2 (R:N) schedule (Figure 5C) and effectively loses rhythmicity under the 28–3:4 (R:N) schedule (Figure 5D). Compared with the alternating schedules with a higher frequency, slowly rotating schedules allow the system to achieve a higher synchronization level during the adaptation process while leading to an internal destabilization, despite allowing the system to achieve a higher maximal *R*_*syn*_ during the adaptation process. The destabilization can potentially bring about an allostatic accumulation of the consequent internal adverse effects of the shift schedules (Burgess, 2007).

**FIGURE 6:**
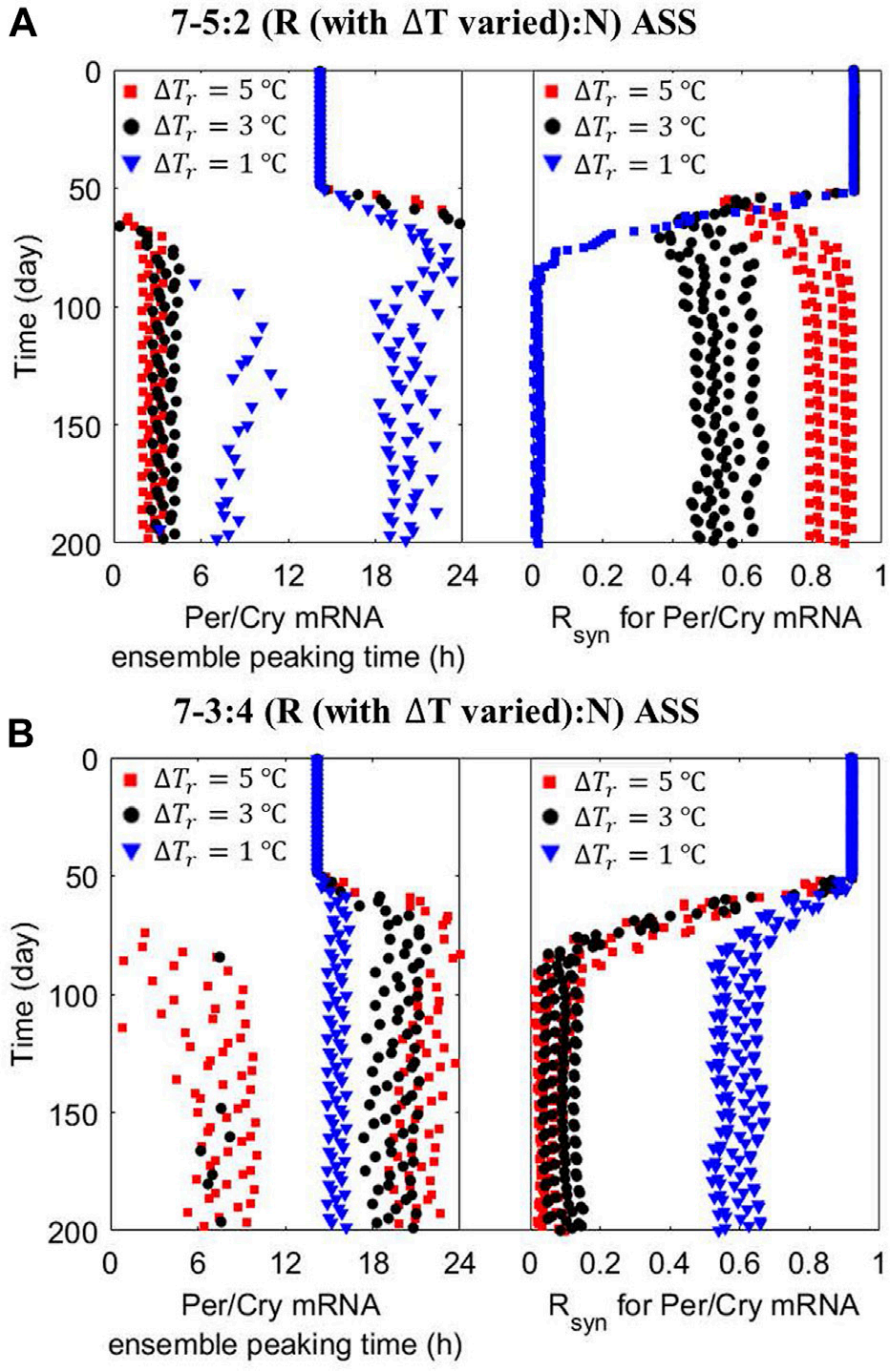
*Zeitgeber’s* amplitude has a schedule-dependent effect on peripheral cells’ nominal ensemble average response to ASSs. The dynamics of the ensemble average phase (left panel) and synchronization level (right panel) of peripheral cell clocks were reevaluated under the **(A)** 7–5:2 (R:N) and **(B)** 7–3:4 (R:N) ASSs with different zeitgeber’s amplitude (ΔT_r_, = 5 (red squares) and 1 (blue triangles) °C) during the reversed temperature pattern. A “good” state of circadian adaptation is characterized by more consistent and robust phase entrainment and higher internal synchrony. Under the 7–5:2 (R:N) ASS, an increase in the amplitude of dominant *zeitgeber* pattern further improves the system’s circadian adaptation, while a decrease destroys it. However, during the 7–3:4 (R:N) ASS, the multicellular system is strongly improved by a decrease in the amplitude of T_r_ pattern, reducing the inherent desynchronizing incline of the system caused by this schedule.

Our early *in silico* experiments identified temperature amplitude and period as critical factors that can be manipulated to affect the circadian alignment of the system. To analyze the impact of amplitude further, we examined it in conjunction with ASSs. In Figure 6, the dynamics of ensemble average phases and *R*_*syn*_ of peripheral clocks are reevaluated under the 7–5:2 and 7–3:4 schedules as a function of ∆T in the $T_{r}$ pattern by examining the cases of high ( $∆T_{r}=5\;℃$ ), nominal ( $\Delta T_{r}=3^{\;o}C$ ) or low ( $∆T_{r}=1\;℃$ ) amplitude. We observe that high $T_{r}$ amplitude strengthens synchronization, while low $T_{r}$ amplitude reduces it for the 7–5:2 ASS (Figure 6A). We predict an increase of *R*_*syn*_ from ∼ 0.5−0.6 (partial synchronization) under nominal $∆T_{r}$ to ∼ 0.8−0.9 (nearly complete synchronization) under high $∆T_{r}$ . In contrast, a decrease to 0 under low $∆T_{r}$ is observed, meaning a loss of the entrainment of *Per/Cry* mRNA occurs when the $T_{r}$ amplitude is decreased substantially. Since the system is inclined to adapt to the characteristics of the most dominant temperature pattern under the 7–5:2 schedule, an increase in the amplitude of dominant z*eitgeber* pattern improves the system’s adaptation, whereas an amplitude decrease weakens it. However, the response is reversed under the 7–3:4 ASS (Figure 6B), where a low-amplitude $T_{r}$ rhythm enhances the entrainment and synchronization of peripheral clocks. This is likely because the 7–3:4 schedule tends to desynchronize the system. Therefore, reducing the impact of any pattern in the perturbed z*eitgeber* schedule diminishes the loss of synchronization.

### Individualized responses to alternating temperature schedules

Recent evidence (Rajaratnam et al., 2013; Rao and Androulakis, 2019; Scherholz et al., 2020) suggests that adaptation to *zeitgeber* characteristics strongly depends on an individual’s ability to sense and be influenced by them. Therefore, we hypothesize that individual peripheral synchronization is driven by, among others, differences in personalized temperature sensing as indicated by $v_{act0,ts1}$ , $v_{act1,ts1}$ and $k_{b,ts2}$ for computational simplicity (see details in Materials and Methods). These parameters are theoretically designed to represent an individual’s temperature-induced activation and subsequent effector response, reflecting the sensitivities of temperature sensing, processing, and transduction that are physiologically crucial for the temperature entrainment event. To test this hypothesis, we sampled these parameters and determined the peripheral synchronization of *Per/Cry* mRNA as a marker for an individual’s internal coherency/circadian adaptation. For the same values of $v_{act0,ts1}$ , $v_{act1,ts1}$ and $k_{b,ts2}$ , we estimated the synchronization levels under a “nominal” temperature schedule (*normal T*, Eq. 16) and three ASSs: 7–6:1 (R:N), 7–5:2, and 7–3:4 (R:N). Maladaptation to alternating *zeitgeber* has potential adverse effects (McGowan and Coogan, 2013). To assess more accurately how personalized ASSs impact synchronization, we performed a multi-linear regression to determine the likely impact of each individualized factor on the entrainment of a population of peripheral cell clocks: $R_{syn}=\beta_{0}+\beta_{1}v_{act0,ts1}+\beta_{2}v_{act1,ts1}+\beta_{3}k_{b,ts2}+\epsilon$ . The results are summarized in Table 2 and indicate that lower sensing sensitivities to temperature magnitude ( $v_{act0,ts1}$ ) generally grant individuals abilities to maintain a higher mean *R*_*syn,ASS*_. In contrast, higher sensing sensitivities to temperature variation ( $v_{act1,ts1}$ ) only contribute for the 7–3:4 (R:N) ASS (also see Supplementary Figure S3).

**TABLE 2:** Multi-linear regression analysis on the impact of individualized factors (*v_act0,ts1_*, *v_act1,ts1_*, *k_b,ts2_*) on peripheral synchronization degree under different ASSs.

*Temperature schedule*	*Nominal (* $T_{n}$ *)*		*7–6:1*		*7–5:2*		*7–3:4*	
*Coefficient*	*value*	*p-value*	*value*	*p-value*	*value*	*p-value*	*value*	*p-value*
$\beta_{0}$	0.904	0	0.800	0	0.495	$10^{-8}$	0.269	$10^{-5}$
$\beta_{1}$	−0.806	$10^{-14}$	−1.676	$10^{-13}$	−4.458	$10^{-16}$	−3.252	$10^{-16}$
$\beta_{2}$	0.089	$10^{-6}$	0.144	$10^{-4}$	0.369	$10^{-4}$	0.623	$10^{-14}$
$\beta_{3}$	0.002	$10^{-4}$	0.007	$10^{-7}$	0.017	$10^{-8}$	−0.006	$10^{-3}$

**FIGURE 7:**
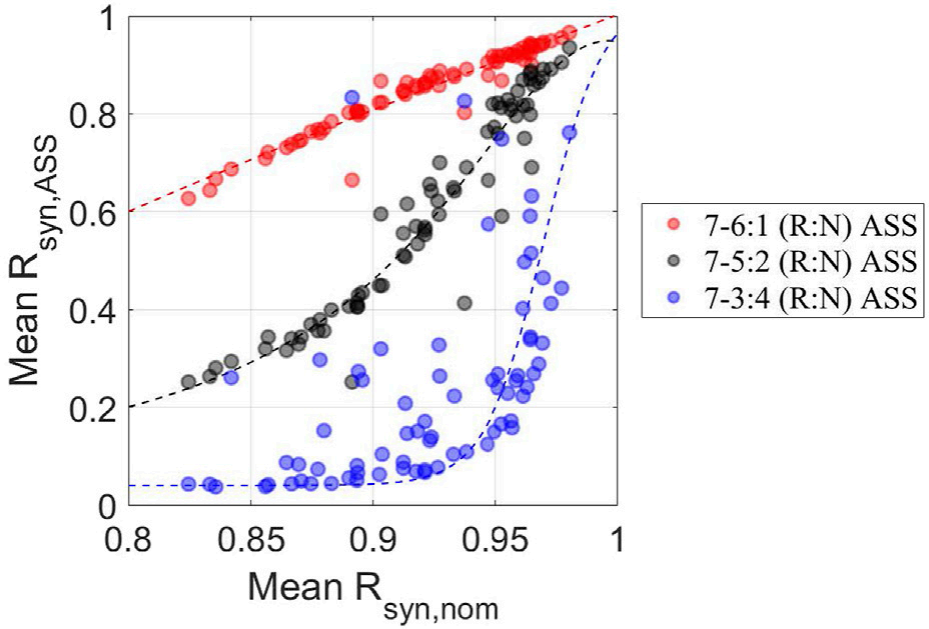
Individualized adaptive performance to ASSs depends on the schedule and the initial homeostatic state The synchrony degree under ASSs (mean *R_syn,ASS_*) was plotted against the homeostatic synchronization state (mean *R_syn,nom_*) for 7–6:1 (red circles), −5:2 (black circles), and −3:4 (blue circles) (R:N) schedules. Consistent with previous observations, individuals tend to exhibit better adaptation to circadian disruptive circumstances, as reflected by overall higher mean Rsyn, Ass when subject to 7–6:1, −5:2 than −3:4 (R:N) schedules. On the other hand, individuals with higher mean Rsyn, nom have higher mean *R_syn,ASS_*, indicating a critical and positive role of the pre-existing level of personalized internal synchronization in determining the personalized performance of circadian adaptation to ASSs.

We then evaluated the individual’s ability to remain entrained under an ASS as a function of the robustness of its circadian rhythms under the nominal temperature schedule by plotting the $R_{syn,ASS}$ vs. $R_{syn,nom}$ (Figure 7). Interestingly, it is observed that the more robust the synchronization before exposure to ASSs, the higher the adaptation to ASSs. Moreover, confirming our earlier observations, the less vulnerable the schedule (7–6:1), the higher the synchronization, while the most susceptible ASS (7–3:4) exhibits a sharp curve of the relationship between the personalized synchronization levels before and after the ASS. Since a phase-maintained, or said, minimal repeated re-entrainment behavior was hypothesized to serve as a long-term mechanism for a subject to prevent adverse consequences of being forced to persistently readapt to alternating patterns of shift work (McGowan and Coogan, 2013), we postulate that a tolerant individual would have a more dynamically stable and higher level of internal synchronization, which is defined by a higher mean $R_{syn,ASS}$ over the entire course of adaptation. Based on this, these results qualitatively capture experimental observations noting that subjects tolerant to long-term shift work are likely to have a more robust homeostatic circadian rhythm (Reinberg et al., 1980).

## Discussion

The synchronization of the internal physiological environment by *zeitgebers* is essential to maintaining homeostasis in living organisms. Although mammalian circadian entrainment/alignment has been predominantly studied both experimentally *(in vivo*, *ex vivo*, *in vitro*) and computationally (*in silico*) using light and feeding as *zeitgebers*, the role of temperature rhythms has received less attention. Temperature rhythms convey entrainment signals to peripheral cells, and their disturbance may lead to phase and period disruptions. Earlier works have presented mathematical models describing temperature entrainment of a single circadian clock in *Neurospora crassa* (Burt et al., 2021). In our study, we proposed a mathematical model representing the sensing and transduction of temperature and its role in entraining an ensemble of peripheral clocks in mammals through a simplified HSR pathway (Figure 1). Our model aims to provide insights into *in vitro* temperature entrainment.

Earlier experimental studies have reported that simulated body temperature rhythms can sustain or reset oscillations of circadian gene expression in cultured fibroblasts (Brown et al., 2002; Saini et al., 2012). In accordance with experimental findings, our model demonstrates that temperature oscillations not only induce and maintain the ensemble average oscillation of a virtual population of cells (Figure 2) but can also act as signals whose strengths determine the onset of circadian rhythms (Figure 3). Our results provide computational support to the potential of temperature cycles for inducing and sustaining periodic oscillation of cellular function in cultured cells or tissues (Abraham et al., 2010; Abraham et al., 2018; Stowie et al., 2019).

The entrainment of oscillatory systems generally depends on both the characteristics of the entraining signal and the intrinsic properties of the entrainee (Burt et al., 2021). To decipher the individual contributions, we first systematically examined the effects of amplitude, magnitude (average), and period of the entraining temperature signal on the synchronization properties of an ensemble of entrained clocks in peripheral cells. Since the synchronization of endogenous rhythms to *zeitgeber* cycles is fundamental for the adaptive function of circadian clocks (Abraham et al., 2010), we characterized both ensemble average phase distribution and synchronization (*R*_*syn*_). The absence of a periodic stimulus or its rhythm ( $k_{hsf1}$ = 0 or ΔT = 0) induces a wide distribution of single cell phases and periods (Figures 4B,C). Therefore, we predict that, peripheral cell oscillators are predicted to have a blunted ensemble average oscillation (Figure 4B) (Balsalobre et al., 1998; Nagoshi et al., 2004; Welsh et al., 2004), meaning a loss of synchronization. This is consistent with *in vitro* experimental observations reporting that either *Hsf1* KO, using HSF1 inhibitor, or exposure to a constant temperature, results in the rhythmicity loss of a cell population. Moreover, strengthening *zeitgeber*’s strength by increasing temperature amplitude can lead to robust entrainment of the cell population, indicating this manipulation can restore circadian rhythmicity (Gachon et al., 2004).

Circadian disruptions induced by alternating *zeitgeber* schedules such as irregular feeding, shift work, and jetlag are associated with an increased risk of chronic diseases, including depression, cancer, cardiovascular disease, metabolic syndrome, and diabetes (Wang et al., 2011). It is hypothesized that circadian misalignment leads to the desynchronization of peripheral clocks and eventually to the dysregulation of downstream processes mediated or regulated by clock genes (Wang et al., 2011; Rajaratnam et al., 2013; Kervezee et al., 2020). Although the alternating schedules have been primarily studied in the context of shift work, the implications of imposing temperature alternating shift schedules (ASSs) are lacking.

In order to better understand the effects of temperature ASSs (alternating frequency between and overall duration of alternative patterns), we computationally determined them with respect to the entrainment of a population of peripheral cells. We simulated the ASSs by repeatedly imposing alternative temperature patterns and denote them as “N-n_1_:n_2_”, meaning the overall period is “N” days, of which “n_1_:n_2_” is the relative time spent in each pattern (Figure A1). For example, “7–5:2” implies that the system experiences the overall pattern repeating every 7 days, during which it is first under one temperature pattern for 5 days and then under the inverted pattern for the next 2 days. The pattern “28–5:2” means that the overall pattern repeats every 28 days, and during each 28-day period, the system spends 20 days in one temperature pattern and 8 days in the inverted (20:8 = 5:2). The inversion of the temperature patterns is motivated by the classic definition of shift work, where the active phase becomes the rest and *vice versa*.

One of the key negative implications of alternating patterns of *zeitgebers* (for example, shift work) is that the circadian system never fully adjusts (Haus and Smolensky, 2006; Goldbeter and Leloup, 2021; Boivin et al., 2022). Qualitatively consistent with “shift work” experience, our results indicate that the system will tend to adopt the phase behavior of the pattern it spends most time in. Figure 5A suggests that, in the long run, the system will adopt the phase corresponding to the pattern presented the longest and be better entrained (i.e., internally synchronized). However, if the ASS does not have a dominating pattern and the alterations appear frequently, the system will lose its ability to maintain the underlying coherency and synchronization (Figure 5B). The situation can be exacerbated if the system spends more (total) time under each alteration in the schedule. In Figures 5C,D, even though the relative amount of time spent in each pattern is the same, because the absolute time is longer, the system is forced to oscillate between different entrainment states and thus never gets to readjust to a new coherent phase.

The nature of circadian adaptation under ASSs depends not only on the phase difference and time spent in each pattern but also on the strength of the entrainer. Interestingly, the strength of the entrainer emphasizes the effect of the ASS. Figure 6A depicts the situation where for the 7–5:2 schedule—a schedule expected to lead to coherent long-term response—the elicited long-term synchronized behavior is accentuated by increasing the amplitude of the dominant temperature pattern. On the other hand, the 7–4:3 ASS, which is expected to drive desynchronization, accentuates this effect as the z*eitgeber*’s strength in any pattern decreases (Figure 6B).

Finally, we aimed to assess individualized homeostatic entrainment and responses to ASSs in general. We concentrated on key control points of sensing and transduction of temperature. By sampling the corresponding parameters, we determined the relations between those critical parameters and entertainment responses under different *zeitgeber* schedules, as well as the abilities of individuals to be entrained during different ASSs given their baseline responses. The parameter associated with an individual’s sensing sensitivity to the magnitude (average) of temperature rhythm ( $v_{act0,ts1}$ ) is found to be closely and inversely correlated to both homeostatic and ASS entrainment performances: the lower the sensitivity, the higher the synchronized state in the periphery (regression output table and Supplementary Figure S3). It is important to note that this result does not effectively conflict with that presented in Supplementary Figure S1, as the designed sampling range of $v_{act0,ts1}$ generated a broader numerical range of the term $v_{act0,ts1}T_{avg}$ in Eq. 2, which includes the range produced by variations in $T_{avg}$ . This made a more comprehensive analysis and therefore a more apparent trend mathematically. The computational results also indicate that individuals whose circadian rhythms are robust tend to maintain robustness of their rhythms when subjected to ASSs (Figure 7). This is an important observation as it may lead to the possible stratification of sub-populations vulnerable to circadian disruption.

Admittedly, the mathematical model developed in this study has several limitations. Our simplified models do not account, among others, for 1) the tissue-specific temperature compensation that might be regulated by a phosphoswitch mechanism controlling the stability of PER/CRY (Narasimamurthy and Virshup, 2017), and 2) mediators like temperature-sensitive proteins other than HSF members that may contribute to the observed residual synchronization of HSF1-deficient cells to temperature rhythms (Tamaru et al., 2011). Nevertheless, it is essential to recognize that the purpose of this model is neither to recapitulate the regulation of core body temperature through thermoregulatory centers in the hypothalamus and the nearly unidirectional control of the circadian component by the SCN (Buhr et al., 2010), nor to use the examined alternating temperature schedules as equivalent to (human) shifwork. Instead, it aims to consider temperature as a likely entrainer of *in vitro* systems and determine whether such a *zeitgeber* could produce cellular responses that mimic circadian disruption’s effects. Our model provides a detailed theoretical framework to describe the circadian entrainment mechanisms of body temperature. The model qualitatively recapitulates critical observations and offers a blueprint for subsequent experiments to validate the induction and disruption of circadian rhythms *in vitro* or *ex vivo* by temperture rhythms. Our work contributes to the development of cell culture systems that can beter represent circadian biology (Mihelakis et al., 2022).

## Conclusion

The early pioneering works of Goodwin, Goldbeter, Tyson, and others demonstrated how autonomous oscillations can result from well-defined networks of mutually regulated components. When subjected to zeitgebers, the entrainment of these “peripheral clocks” follows from the basic mathematical concepts describing forced oscillators. The question, however, concerns how *zeitgeber* information is transduced via appropriate signaling mechanisms to engage, entrain, or disrupt the innate circadian rhythms. We aimed to express how “generalized” structures materialize in a specific context to convert findings into actions. This work had two broader aims: 1) it is an attempt to shed some light on how T exerts its entraining actions. The fact that T can act as a *zeitgeber*, endowed with all the well-established properties of a zeitgeber, is expected. However, how this materializes is still being explored. Knowing this would be vital as we move to the next step, which is understanding how multiple zeitgebers convey their entraining signals; 2) one of the main issues is the lack of tunable *in vitro* systems that would enable to reproduce conditions of circadian disruption in a well-controlled environment. Motivated by early experimental observations, we further argue the importance of T to induce sustained and tunable oscillations. This would be critical to assess the role of circadian rhythms in physiology and pharmacology in a controlled environment. Cell culture systems are limited in generating, sustaining, and disrupting circadian rhythms at will to study their broader physiological implications in a controlled environment. The continuous efforts to assess the role of circadian rhythms and the need to reduce animal testing necessitates the development of appropriate surrogates to provide a more relevant environment. Recent work has demonstrated that temperature is a potent *in vitro* entrainer of peripheral clocks. In this direction, we developed a mathematical model that integrates temperature sensing, transduction, and an HSF1-mediated signaling component of the HSR pathway asnd peripheral clock genes to describe the temperature entrainment process in a multicellular system.

By investigating unperturbed and perturbed circadian conditions and considering individualized temperature signaling function, our model suggests an amplitude and/or period-dependent temperature effect on the entrainment of cellular clocks. We further showed how alternative temperature rhythms could mimic conditions of circadian disruption and their implications. Our results demonstrate that temperature rhythm can serve as an *in vitro* analog of circadian entrainment and an experimental tool to study the implications of circadian disruption. Since our model incorporates essential components and possible pathways of the temperature entrainment process, it provides the foundations for further experimental system and model development.

## Data Availability

The original contributions presented in the study are included in the article/Supplementary Material, further inquiries can be directed to the corresponding author.
