# Supplementary figures and images for: Mathematical modeling of temperature-induced circadian rhythms
Source: Front Syst Biol. 2024 Mar 25;4:1256398. doi: 10.3389/fsysb.2024.1256398 (PMC12341955; doi:10.3389/fsysb.2024.1256398)

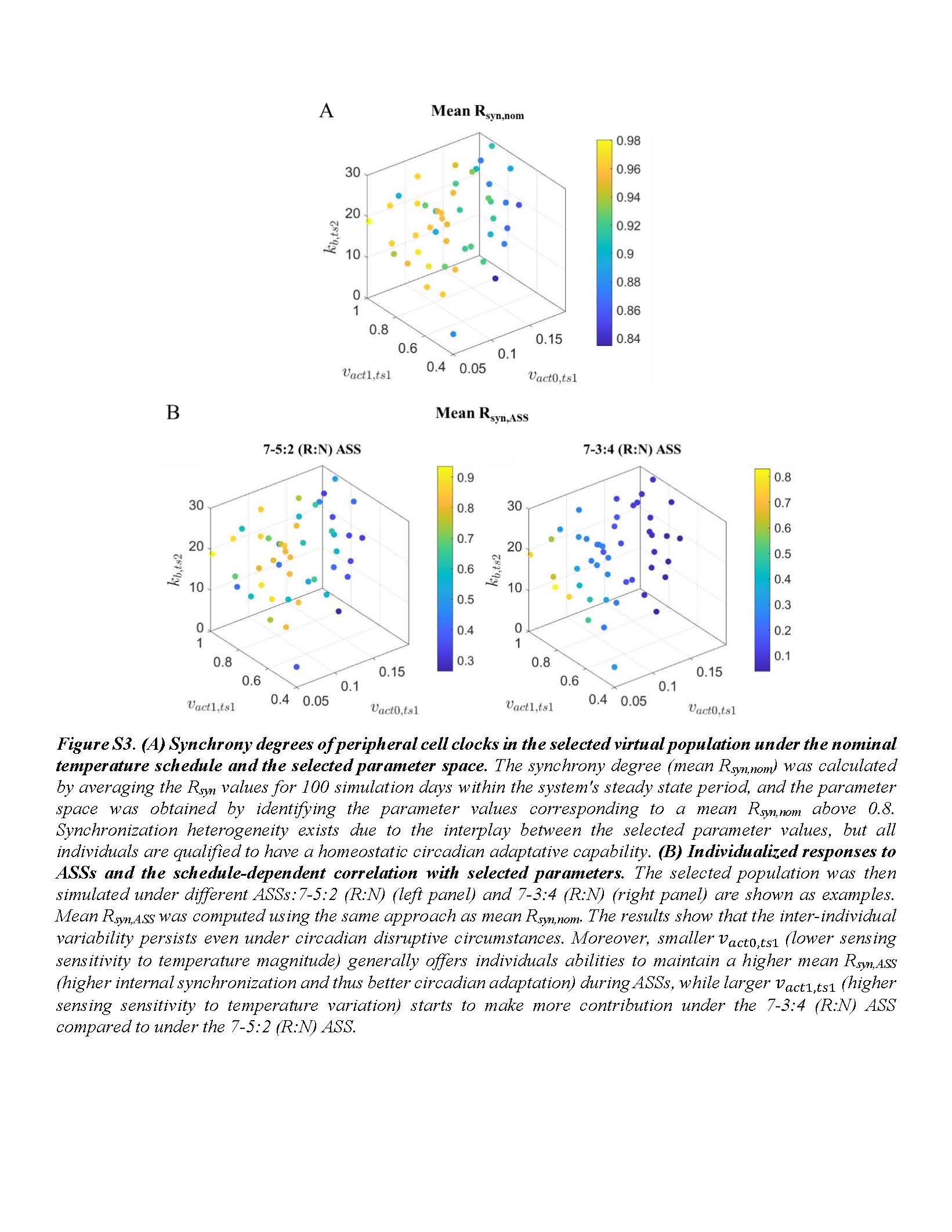

Supplement: Supplementary file 1 [file Image3.JPEG]

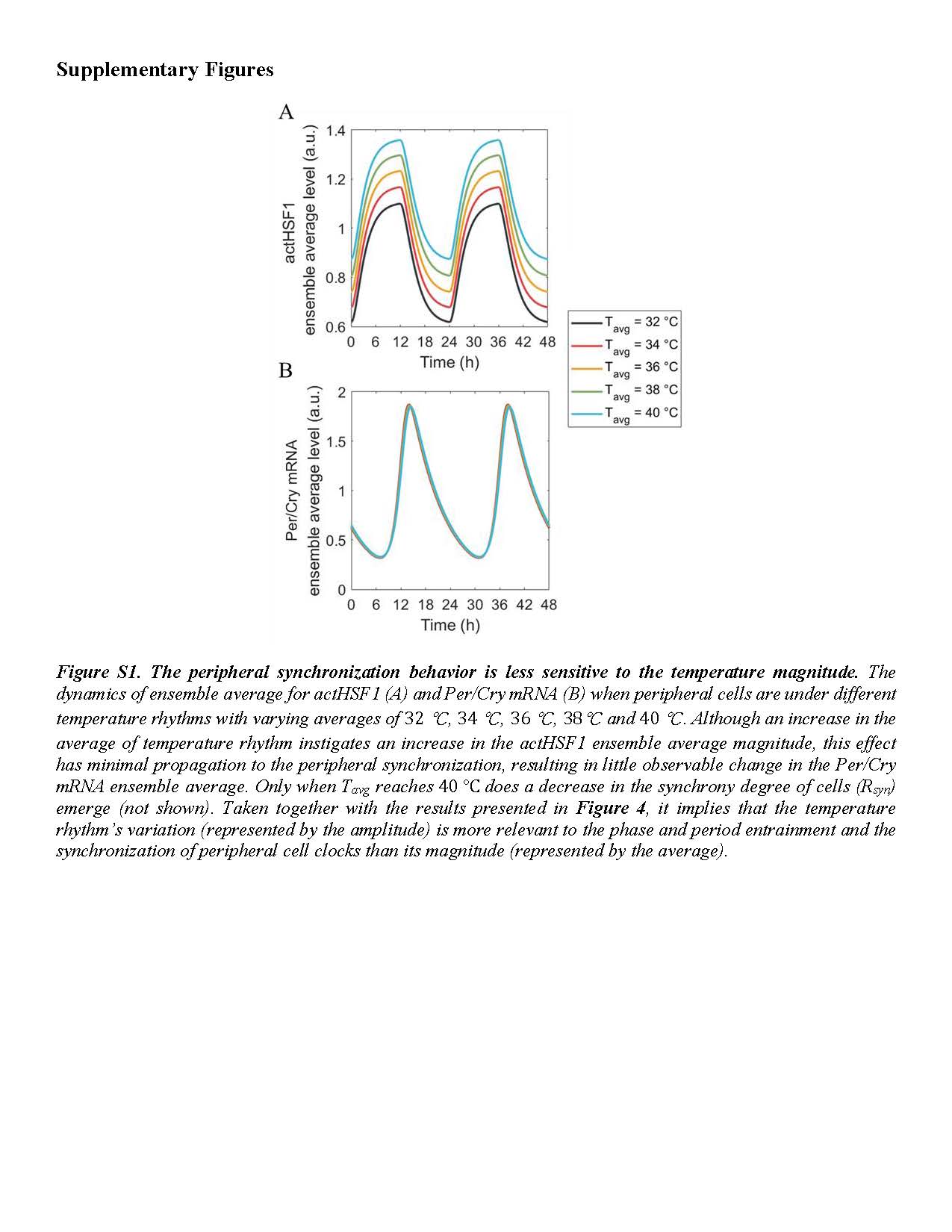

Supplement: Supplementary file 2 [file Image1.JPEG]

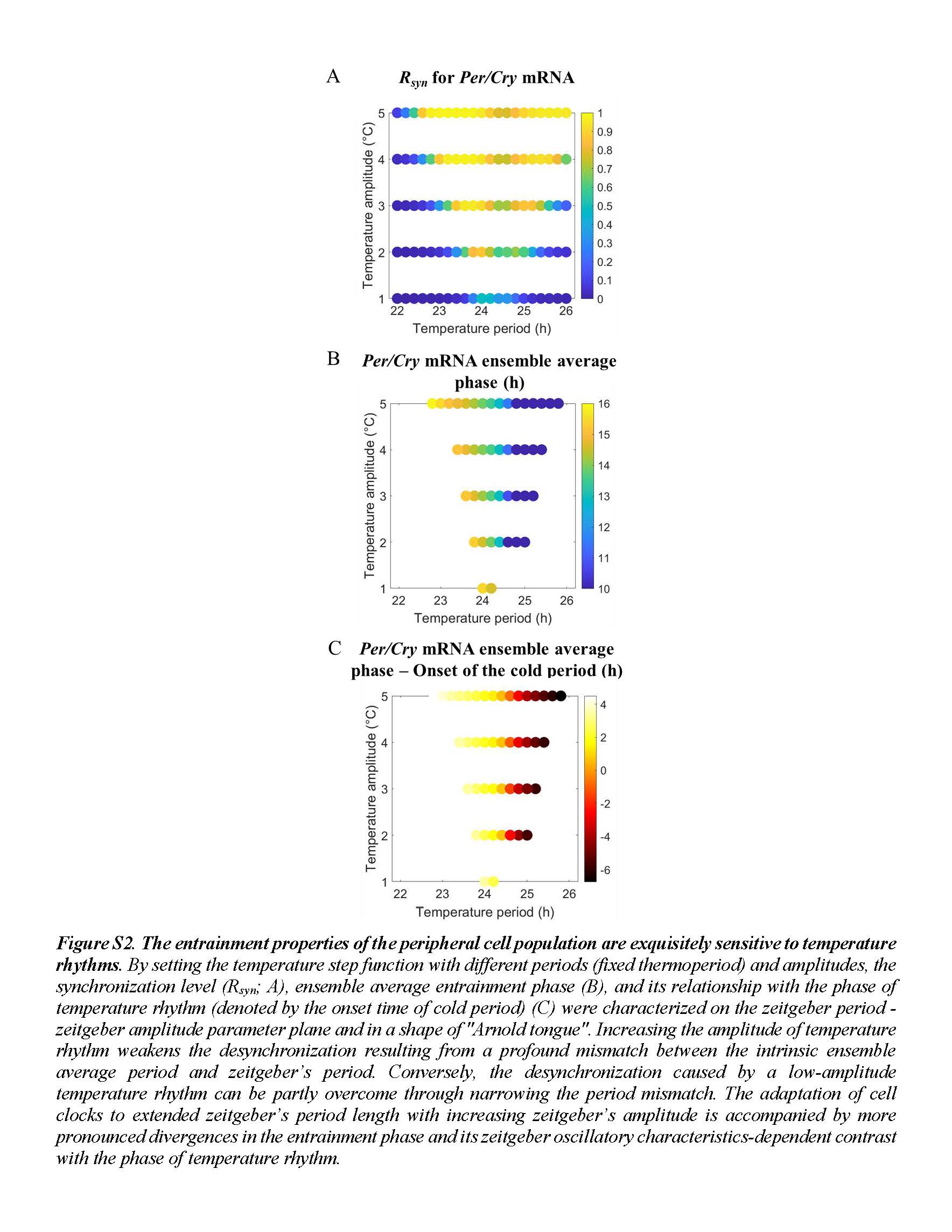

Supplement: Supplementary file 3 [file Image2.JPEG]
